# 

**Published:** 1850-05

**Authors:** 


### NOTICE TO READERS AND CORRESPONDENTS.

Original communications have been received from Drs. J. J. Lescher, V. M. Saterlee, and W. Matthews.

*Injections of Iodine and Iodide of Potassium in Spina Bifida and Hydrocephalus.*—Dr. Brainard has employed the injection, during the past year, in three additional cases of *Spina Bifida,* with satisfactory results; and in one case of chronic congenital *Hydrocephalus.* This latter case has been six months under treatment; during wich time the operation has been repeated twenty one times. As large an amount as gr. xij of Iodine, and gr. xxxvj Iodide of Potass to oz. j of Distilled Water, has been injected into the head, at one operation, without producing inconvenient results. A full report of the cases will be furnished to our readers in a future number.

We regret that a notice of Maclise's Surgical Anatomy was overlooked in sending copy to the compositor. It shall appear in our next.

We have been compelled to abridge the obituary notice of Dr. Pratt, of Ottowa, as there has been a demand on our pages, for matter of more general interest to our readers. We may as well take this time to request our correspondents to make their communications on such subjects—and indeed all subjects, as concise as the nature of the subject will admit. We aim to give as great a variety as possible; and to accomplish this in our narrow limits, requires that we should apply the condensing process to many of the communications furnished us.

We shall hereafter add a new department to the Journal, in which we will give a summary of the more interesting articles in the American and Foreign Journals; as the large amount of original matter furnished us prevents us from extracting many articles entire.

In addition to the publications already noticed, we have received the following:—Report of the Pennsylvania Hospital for the Insane, for 1849. Seventh Annual Report of the Managers of the State Lunatic Asylum of New York.—Report of the Ohio Lunatic Asylum.—Valedictory Address to the graduating Class in the Medical Department of Transylvania University. by Prof. Boling.—Lecture introductory to the course of Surgery, delivered at the Mass. Med. College, in Boston, by Prof. Bigelow. Catalogue of Starling Medical Department of Transylvania University.—Comparative value of the different Anæsthetic Agents: by Geo. Hayward M.D.—Researches on the Natural History of Death, by Bennett Dowler, M.D.—Dental News Letter.—Annual Report of Physicians to the Marine Hospital. (From the Author, Dr. F. C. Stewart.)

We have been furnished by Messrs. J. H. Reed & Co., with a specimen of a new remedy for Epilepsy, (the Extract of *Cotyledon Umbilicus*), mentioned with approbation in No. xix of Braithwaites Retrospect.

### LIST OF RECEIPTS FOR MARCH & APRIL, 1850.

### ILLINOIS.

| | |
|---|---|
| Drs. C. L. Webster, | $5 00 |
| Jos. Tefft, | 2 00 |
| W. L. Gray, | 1 00 |
| R. B. Landon, | 1 00 |
| S. F. Hume, | 6 00 |
| A. W. Bowen, | 6 00 |
| A. D. Wilson, | 4 00 |
| O. S. Johnson, | 2 00 |
| J. Brinckerkoff, | 2 00 |
| J. V. Z. Blaney, | 4 00 |
| E. Farmer, | 3 00 |
| H. H. Hinman, | 1 00 |
| C. J. Miller, | 2 00 |
| C. M. Hamilton, | 2 00 |
| I. H. Brown, | 2 00 |
| Jesse Bennett, | 2 00 |
| L. A. Winslow, | 4 00 |
| A. E. Shepherd, | 2 00 |
| John Lecrone, | 2 00 |
| Ed. Moore, | 3 00 |
| C. H. Duck, | 2 00 |
| P. Kirwan, | 2 00 |
| P. B. McKay, | 4 00 |
| G. W. Wentworth, | 2 00 |
| R. E. Bennett, | 4 00 |
| A. Berchleman, | 2 00 |
| T. Leeds, | 2 00 |
| H. Fisk, | 1 00 |
| E. S. Cooper, | 3 00 |

### INDIANA.

| | |
|---|---|
| Drs. I. B. Hedges, | $2 00 |
| B. Bartholomew. | 2 00 |
| Maulsby & Robins, | 2 00 |
| D. B. Crouse, | 4 00 |
| Joseph Emmons, | 2 00 |
| C. C. Garrett, | 2 00 |
| Jerome Hoover, | 4 00 |
| Nathan Roberts, | 2 00 |
| G. A. Moss, | 4 00 |
| W. W. Doherty, | 2 00 |
| J. W. Green, | 5 00 |
| U. B. Tingley, | 2 00 |
| Jno. A. Wood, | 2 00 |
| J. M. Bell, | 2 00 |
| E. H. Drake, | 4 00 |
| J. H. Buell, | 2 00 |
| J. Pennington, | 4 00 |
| John Sims, | 4 00 |
| Benj. F. Russell, | 2 00 |
| M. Bell, | 2 00 |
| C. F. McNeill, | 4 00 |

### MICHIGAN.

| | |
|---|---|
| Drs. S. D. Richardson, | $2 00 |
| L. Tompkins, | 2 00 |
| A. B. Palmer, | 2 00 |
| H. Lockwood, | 3 00 |
| P. H. Bowman, | 4 00 |
| Henry Davis, | 3 00 |
| A. DeArmand, | 2 00 |

### WISCONSIN.

| | |
|---|---|
| Drs. Sol. Blood, | $2 00 |
| L. J. Barrows, | 1 00 |
| J. R. Bradway, | 2 00 |
| S. Spencer, | 6 00 |
| P. McBrien, | 2 00 |
| I. R. Rood, | 1 00 |
| Jas. Cody, | 2 00 |
| B. F. Collins, | 2 00 |

### IOWA.

| | |
|---|---|
| Drs. Wier & Beck, | $2 00 |
| J. Doron, | 2 00 |

### OHIO.

| | |
|---|---|
| Dr. A. B. Ferris, | $2 00 |

### NEW YORK.

| | |
|---|---|
| Albany Medical College, | $2 00 |

### CALIFORNIA.

| | |
|---|---|
| Dr. H. T. Brown, | 2 00 |

---

### VACCINE VIRUS

Can be obtained by sending One Dollar, post-paid, to the address of the Editors of this Journal. The Virus will be enclosed in a letter, and sent by the return mail.

PROSPECTUS OF THE THIRD VOLUME OF

### THE NORTH-WESTERN MEDICAL AND SURGICAL JOURNAL.

---

EDITED BY

JOHN EVANS, M.D.,

PROFESSOR OF OBSTETRICS AND DISEASES OF WOMEN AND CHILDREN IN RUSH MEDICAL COLLEGE, AND

EDWIN G. MEEK, A.M, M.D.

---

This work, as heretofore, will be issued at Chicago and Indianapolis, on the first of every other month, beginning with May, in numbers, each containing from 88 to 96 pages, making at the end of the year, a volume of about 550 pages, octavo, with title page and index complete.

In addition to the usual large amount of original matter, the forth coming volume will contain a

HISTORY OF THE PROFESSION IN THE UNITED STATES,
*From the Earliest Settlement of the Colonies, to the Present Tim[illegible].*
BY PROF. N. S. DAVIS, M.D.

Which of itself will be richly worth the subscription price. No physician who properly regards his profession should fail to secure a copy.

As the list of contributors to the work is annually increasing, it may be expected to furnish as good a variety of interesting original matter as any work of the kind in the country. The diseases of the west will claim especial attention.

Contributions to our pages, especially from physicians of the North-west, are earnestly solicited.

TERMS.---Two Dollars per annum, *in advance*, which may be paid to any of our authorized agents, or sent to us by mail, post paid, at our risk, directed to the "North-Western Medical and Surgical Journal, Chicago, Illinois." If payment be delayed until the close of the volume, three dollars will be invariably charged. No subscription received for less than an entire volume.

A few copies of the FIRST and SECOND VOLUMES remain unsold, and will be sent by mail for **ONE DOLLAR PER. VOLUME;** (the cash to accompany a post-paid order,) or the work will be furnished, half-bound, in sheep, for $1 25 per volume.

☞ A list of receipts will be published in each number.

☞ *Travelling Agents.*—L. D. Latimer, S. A. Pease, C. A. Swan, Edmund Spotswood, A. Loftin, M. O. Crooks, ——— Bennett.

